# Suitability of *R. pulmo* Jellyfish-Collagen-Coated Well Plates for Cytocompatibility Analyses of Biomaterials

**DOI:** 10.3390/ijms24033007

**Published:** 2023-02-03

**Authors:** Yanru Ren, Luo Liu, Xin Xiong, Rumen Krastev, Ralf Smeets, Denis Rimashevskiy, Reinhard Schnettler, Said Alkildani, Steffen Emmert, Ole Jung, Mike Barbeck

**Affiliations:** 1 Clinic and Policlinic for Dermatology and Venereology, University Medical Center Rostock, 18057 Rostock, Germany; 2 Beijing Advanced Innovation Center for Soft Matter Science and Engineering, College of Life Science and Technology, Beijing University of Chemical Technology, Beijing 100013, China; 3 NMI Natural and Medical Sciences Institute, University of Tübingen, 72770 Reutlingen, Germany; 4 Faculty for Applied Chemistry, Reutlingen University, Alteburgstraße 150, 72762 Reutlingen, Germany; 5 Department of Oral and Maxillofacial Surgery, University Medical Center Hamburg-Eppendorf, 20246 Hamburg, Germany; 6 Department of Oral and Maxillofacial Surgery, Division of Regenerative Orofacial Medicine, University Medical Center Hamburg-Eppendorf, 20246 Hamburg, Germany; 7 Department of Traumatology and Orthopedics, Peoples’ Friendship University of Russia, 117198 Moscow, Russia; 8 University Medical Centre, Justus Liebig University of Giessen, 35390 Giessen, Germany; 9 BerlinAnalytix GmbH, 12109 Berlin, Germany

**Keywords:** jellyfish collagen, cytocompatibility, collagen coating, well plate, in vitro, medical devices, biomaterials

## Abstract

Cytocompatibility analyses of new implant materials or biomaterials are not only prescribed by the Medical Device Regulation (MDR), as defined in the DIN ISO Norm 10993-5 and -12, but are also increasingly replacing animal testing. In this context, jellyfish collagen has already been established as an alternative to mammalian collagen in different cell culture conditions, but a lack of knowledge exists about its applicability for cytocompatibility analyses of biomaterials. Thus, the present study was conducted to compare well plates coated with collagen type 0 derived from *Rhizostoma pulmo* with plates coated with bovine and porcine collagen. The coated well plates were analysed in vitro for their cytocompatibility, according to EN ISO 10993-5/−12, using both L929 fibroblasts and MC3T3 pre-osteoblasts. Thereby, the coated well plates were compared, using established materials as positive controls and a cytotoxic material, RM-A, as a negative control. L929 cells exhibited a significantly higher viability (#### *p* < 0.0001), proliferation (## *p* < 0.01), and a lower cytotoxicity (## *p* < 0.01 and # *p* < 0.05)) in the Jellagen® group compared to the bovine and porcine collagen groups. MC3T3 cells showed similar viability and acceptable proliferation and cytotoxicity in all collagen groups. The results of the present study revealed that the coating of well plates with collagen Type 0 derived from *R. pulmo* leads to comparable results to the case of well plates coated with mammalian collagens. Therefore, it is fully suitable for the in vitro analyses of the cytocompatibility of biomaterials or medical devices.

## 1. Introduction

Cell culture systems are of value in testing the biocompatibility of medical devices and biomaterials before they are introduced into further preclinical in vivo trials and clinical studies. In recent years, in vitro methods for assaying biomaterials have gained importance owing to the growing concern over the use of animals for testing biomaterials. Significant effort is therefore being focused toward developing predictive and quantitative—but also simple and reliable—methods of testing using cultured cells. At present, a number of methods for measuring both the cytotoxicity and the specific cytocompatibility of different materials are available [1,2,3]. The usefulness of these systems is no longer confined to screening new materials as they can be used to study the mechanisms of action of cell or tissue/material interactions of various biomaterials. 

Collagen has long been considered an ideal substrate for culturing cells [4,5,6]. It accounts for more than 25% of total mammalian protein and provides structural and strength support for the body as a major component of the extracellular matrix [7,8]. Moreover, collagen is naturally biologically active as an endogenous component of the body to optimally mimic the in vivo environment [9]. Collagen has been shown to be involved in the regulation of cell adhesion, proliferation, and differentiation by binding to at least five different receptors (integrins, DDR, Glycoprotein VI, Osteoclast-associated receptor (OSCAR), LAIR-1, and uPARAP/Endo180) [9,10]. This is crucial for in vitro cell culture because all cells from normal tissues are anchorage-dependent, except for cells from the hematopoietic system and tumor cells [11]. In other words, cells must be anchored to an extracellular-matrix-like substrate and interact with their surroundings to grow and proliferate normally, otherwise they will experience growth arrest and anoikis [12]. The natural pores of collagen also support the attachment and migration of cells on its surface, which is essential for the normal physiological activities of cells [13].

For decades, cows and pigs have been the main sources of heterologous bovine and porcine collagen. This is largely influenced by human meat consumption habits [4,14]. However, both sources of collagen face limitations due to infectious diseases (e.g., bovine spongiform encephalopathy, foot-and-mouth disease, and swine flu) and religious factors [4]. In this case, additional evaluation and purification procedures are necessary due to safety concerns, which lead to higher costs and face the challenge of preserving the “natural” collagen structure during the complex purification process [15]. In addition, as complex organisms, differences in the origin, age, sex, and size of mammals may cause inevitable inconsistencies between different batches of collagen, which can sometimes be detrimental to the research [4,16,17]. Therefore, a more reliable, alternative source of collagen is urgently sought.

Jellyfish-derived collagen has gradually entered the limelight in recent years [8,18]. As ancient invertebrates, jellyfish have a simple physiological structure consisting mainly of water and a collagen-rich mesoglea [19]. Jellyfish-derived collagen is homogeneous with mammalian types I, II, III, V, and IX, and is therefore also known as “type 0 collagen” [20]. The desirability of jellyfish-derived collagen as an emerging alternative source is based on (1) the avoidance of complex mammalian tissues, (2) its low cost and improved carbon footprint, (3) fewer infectious disease and religious concerns, (4) batch-to-batch consistency, and (5) benefits for marine waste utilization and ecological management [5,21,22]. However, it should be noted that jellyfish collagen is less cross-linked than mammalian collagen due to its lower proline and hydroxyproline content, which makes jellyfish collagen products face stability concerns [19].

The jellyfish species currently available for collagen extraction are *Somolophus meleagris* jellyfish [22,23], *Rhizostomous* jellyfish [24], and *Chrysaora sp.* jellyfish [16]. Among these species, collagen from *Rhizostoma pulmo* has been shown to have a sequence homology with the vertebrate collagen type I [19]. The biocompatibility of jellyfish collagen has been confirmed by many studies, including cytotoxicity, adhesion tests, and the secretion of pro-inflammatory factors [19,22,24,25]. Jellyfish collagen is thought to elicit cellular responses similar to mammalian collagen involving adhesion, proliferation, and migration [19]. In addition, jellyfish collagen has been shown to induce a long-term, anti-inflammatory macrophage response as well as excellent vascularization in vivo to support bone tissue regeneration [26,27].

The Jellagen^®^ collagen from *R. pulmo* (JC) involved in this study has been reported to have potential in culturing human iPSC-derived Microglia (iMGL) and ovarian cancer (OvCa) cell lines [24,28]. In this context, Jellagen^®^ collagen is of interest as a culture medium for a cytocompatibility analysis, which is an extensively used tool in the evaluation of biomaterials. Our previous studies have demonstrated that fibroblasts and osteoblasts exhibit good proliferation behaviour on Jellagen^®^-collagen-coated multi-well culture plates and jellyfish 3D scaffolds [25]. However, it remains unclear whether Jellagen^®^ collagen is comparable to porcine- and bovine-derived collagen as a cell culture substrate for in vitro biocompatibility analyses.

In the present study, we report a comparison of well plate coatings based on Jellagen^®^ collagen and bovine and porcine collagen to assess their effects on culturing L-929 fibroblasts and MC3T3 pre-osteoblasts. Cell viability (XTT assay), cytotoxicity (LDH assay), and cell proliferation (BrdU assay) analyses were combinatorially conducted to evaluate the usability of the collagen coating for cytocompatibility analyses. Supplemental LIVE/DEAD staining was used to visualize the growth status of L-929 on well plates with different collagen coatings. The aim of this study was to assess the suitability of Jellagen^®^ collagen as a culture matrix for in vitro biocompatibility analyses in comparison with porcine- and bovine-derived collagen.

## 2. Results

### 2.1. Cytocompatibility Results—L929-Fibroblasts

**Figure 1:**
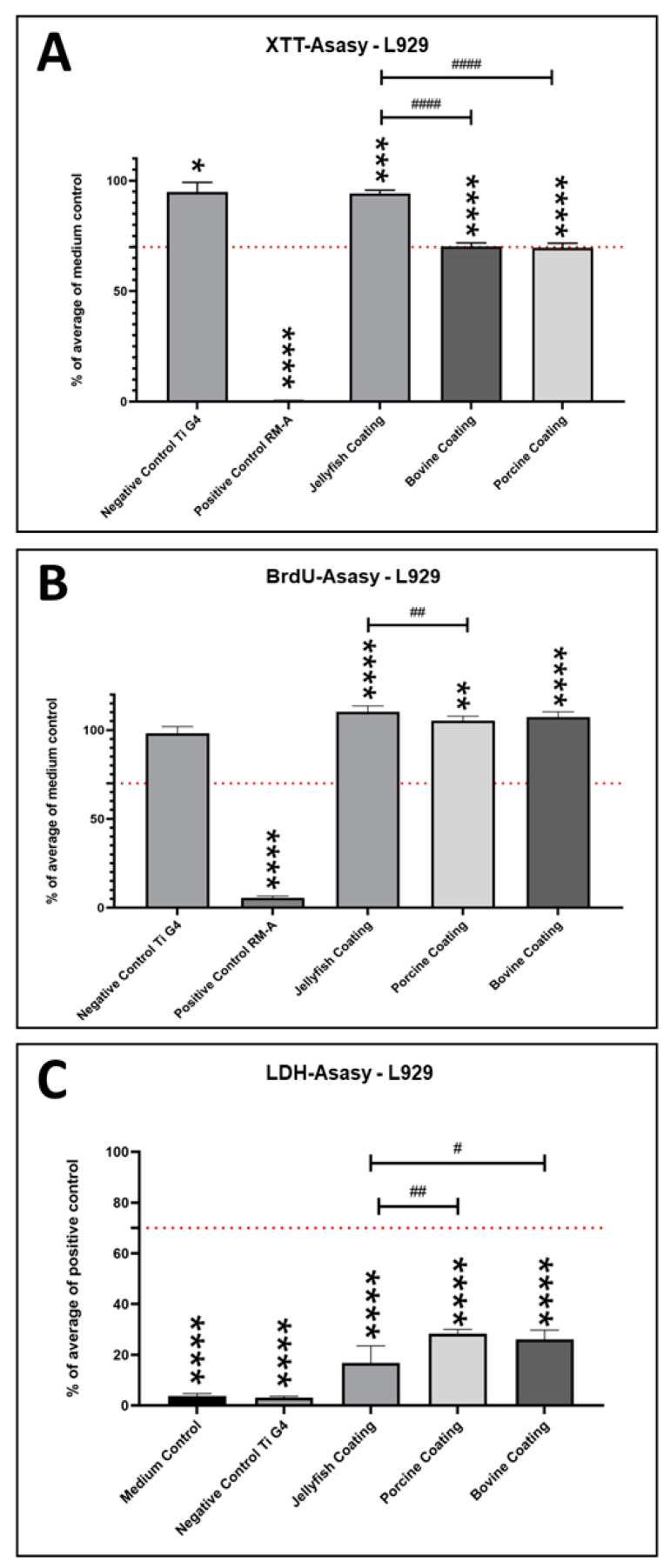
Cytocompatibility results of the different coatings using L929 fibroblasts. (**A**) Viability measured by the XTT assay, (**B**) proliferation measured by the BrdU assay, and (**C**) cytotoxicity measured by the LDH assay. Dotted lines show the respective thresholds that should not be exceeded (LDH) or undershot (XTT, BrdU). Significant differences: #/* *p* < 0.05, ##/** *p* <0.01, *** *p* < 0.001, ####/**** *p* < 0.0001).

The analysis of the cell viability of the L929 fibroblasts via XTT assay revealed that the coating using the Jellagen^®^ collagen induced a significantly lower (*** *p* < 0.001) fibroblast viability compared to the medium control (Figure 1A). Moreover, both the porcine and the bovine coatings showed even significantly lower values (**** *p* < 0.0001) (Figure 1A). Thus, the viability values based on the Jellagen^®^ collagen coating were significantly higher (#### *p* < 0.0001) when compared to the porcine and the bovine coatings (Figure 1A). Altogether, the viability values in the group of the Jellagen® collagen coating were well above the 70% limit defined by the DIN ISO standard, while the values in the other two groups were close to this limit. The XTT analysis also showed that the viability values in the group of the negative control (titanium, grade 4) were significantly lower (* *p* < 0.05) when compared to the medium control. Additionally, the positive control material, RM-A, induced a significantly lower (**** *p* < 0.0001) cell viability when compared to the medium control (Figure 1A).

The proliferation analysis of the L929 fibroblasts via BrDU assay showed that all collagen coatings induced significantly higher values (**p < 0.01and **** *p* < 0.0001) compared to the medium control (Figure 1B). Additionally, the proliferation values in the group of well plates coated with Jellagen^®^ collagen were significantly higher (## *p* < 0.01) when compared to the values in the group with the porcine collagen coating, while no significant difference was found when compared to the values in the group with the bovine collagen coating (Figure 1B). Additionally, the BrDU analysis confirmed that the negative control (titanium, grade 4) showed no significant difference compared to the medium control, while the positive control material, RM-A, induced a significantly lower (**** *p* < 0.0001) cell proliferation value when compared to the medium control (Figure 1B).

Finally, the cytotoxicity analysis via LDH assay revealed that the values in all coating groups and in both control groups, i.e., the medium control group and the group with the grade 4 titanium material, were significantly lower (**** *p* < 0.0001) when compared to the values of the positive control group (RM-A) (Figure 1C). Furthermore, the values in the group with the Jellagen® collagen coating were significantly lower (## *p* < 0.01 and # *p* < 0.05) when compared to the values in the groups with the porcine and bovine collagen coatings (Figure 1C). 

**Figure 2:**
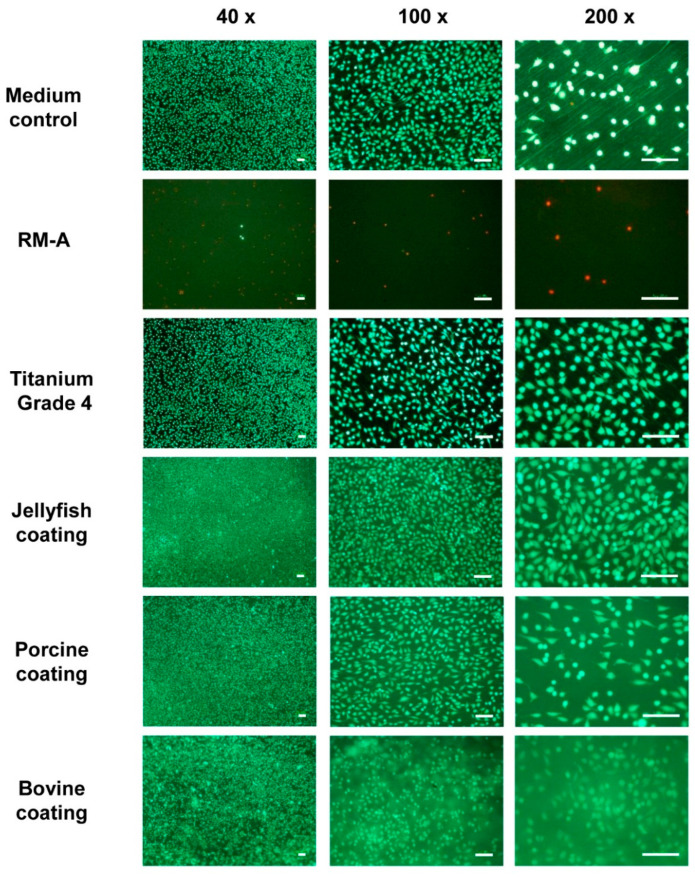
LIVE/DEAD-staining of the controls (rows 1–3) and test samples (rows 4–6). Green: viable cells; red: dead cells (**left column**: 4× magnifications; middle column: 100× magnifications, **right column**: 200× magnifications; scale bars = 100 μm).

The LIVE/DEAD staining results additionally confirmed the data via the afore mentioned assays (Figure 2). The cells in the medium control were, for the very most part, viable, and formed a dense layer with a partial, spindle-shaped form, with only a few dead cells detectable (Figure 2). In the case of the cytotoxic control (RM-A), only a few cells were detectable, of which a few cells were viable, and the majority showed signs of acute cell death (Figure 2). In case of the cytocompatible control material (titanium), a denser layer formation of the L929 fibroblasts was observed, and more cells showed a spindle-like form (Figure 2). In the groups with the Jellagen® collagen coating and the bovine collagen coating, an even higher cell density was detected, and most of the L929 fibroblasts demonstrated a spindle-like form. In the group with the porcine collagen coating, a slightly lower cell density was found, while most of the fibroblasts also showed a spindle-like shape (Figure 2).

### 2.2. Cytocompatibility Results—MC3T3-Pre-osteoblasts

**Figure 3:**
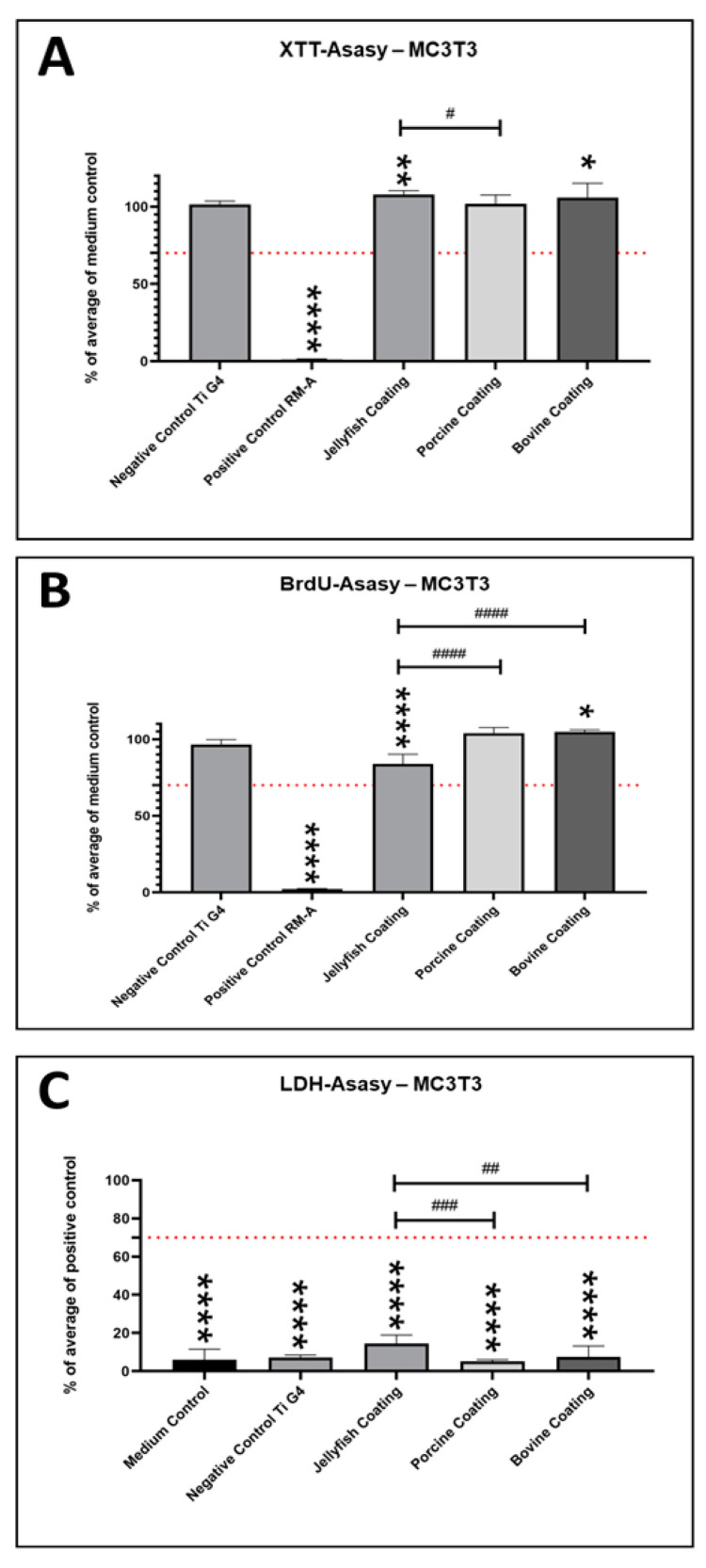
Cytocompatibility results of the different coatings using MC3T3 pre-osteoblasts. (**A**) Viability measured by the XTT assay, (**B**) proliferation measured by the BrdU assay, and (**C**) cytotoxicity measured by the LDH assay. Dotted lines show the respective thresholds that should not be exceeded (LDH) or undershot (XTT, BrdU). Significant differences: #/* *p* < 0.05, ##/** *p* <0.01, ### *p* < 0.001, ####/**** *p* < 0.0001).

The analysis of the cell viability of the MC3T3 pre-osteoblasts via XTT assay showed that both the bovine and Jellagen^®^ collagen coatings induced a significantly higher (* *p* < 0.05 and ** *p* < 0.01) viability compared to the medium control, while no differences were found between the values in the group with the porcine coating and the medium control (Figure 3A). Moreover, the viability values in the group of the Jellagen^®^ collagen coating were significantly higher (# *p* < 0.05) when compared to the porcine coating but not to the values in the group with the bovine coating (Figure 3A). The XTT analysis confirmed that the viability values in the group of the negative control (titanium, grade 4) were comparable to the medium control. Additionally, the positive control material, RM-A, induced a significantly lower (**** *p* < 0.0001) cell viability when compared to the medium control (Figure 3A).

The proliferation analysis of the MC3T3 pre-osteoblasts via BrDU assay showed that only the bovine collagen coating induced significantly higher values (*p < 0.05) compared to the medium control, while no differences were found between the values in the group of the porcine coating and the medium control (Figure 3B). Additionally, the proliferation values in the group of well plates coated with Jellagen^®^ collagen were significantly lower (**** *p* < 0.0001 and #### *p* < 0.0001) when compared to the values in the group of the medium control and the coatings with porcine and bovine collagen (Figure 3B). The BrDU analysis also confirmed that the negative control (titanium, grade 4) and the porcine coating were comparable to the medium control, while the positive control material, RM-A, induced a significantly lower (**** *p* < 0.0001) cell proliferation compared to the medium control (Figure 3B).

Finally, the cytotoxicity analysis via LDH assay revealed that the values in all coating groups but also in the both control groups, i.e., the medium control group and the group with the grade 4 titanium material, were significantly lower (**** *p* < 0.0001) when compared to the values in the group of the positive control (RM-A) (Figure 3C). Furthermore, the values in the group with the Jellagen^®^ collagen coating were significantly higher (## *p* < 0.01 and ### *p* < 0.001) compared to the values in the group of the porcine and bovine collagen coatings (Figure 3C).

## 3. Discussion

In vitro cell culturing is not an easy task. The normal physiological activities of most human cells depend on the three-dimensional network of the extracellular matrix, which provides structural and physiological support for the cells [29]. Therefore, simulating a similar physiological environment in vitro is crucial for a successful cell culture. Well plates are the most widely used tools for in vitro cell experiments, especially in the fields of biomaterials and tissue engineering. Proper surface treatment for well plates is necessary prior to cell inoculation due to their hydrophobic polystyrene surface [30]. As the dominant structural component of the extracellular matrix, collagen provides natural bioactivity to well plates [31]. Collagen surfaces naturally possess ligands that bind to a variety of receptors to regulate cell adhesion, proliferation, and migration [10,13,19]. In addition, collagen substrates support cells in producing their own extracellular matrix during growth [13,32]. Not surprisingly, collagen matrices have become the matrix of choice for in vitro cell cultures. 

The collagen currently utilized for cell cultures is mainly derived from mammalian sources such as bovine, porcine, and murine [6,11]. As the most abundant protein, collagen is widely represented throughout the tissues in the body, such as the skin, pericardium, cartilage, and tendons, which can be easily obtained from slaughterhouse by-products [4,14,33,34]. The manufacturing process to obtain collagen is quite complex due to the structural and compositional complexity of mammalian tissues [17]. However, even if every step of the process is strictly regulated, batch-to-batch inconsistencies can occur due to individual mammalian variability [35]. In addition, current purification methods are also considered insufficient to eliminate the risk of disease infection, such as bovine spongiform encephalopathy (BSE) [36]. However, researchers desire a consistent, disease-free substrate that provides an optimal protocol for in vitro cell culturing.

Jellyfish collagen, derived from marine animals with a higher economic efficiency, is expected to provide an optimized approach to this gap. Unlike mammalian collagen, Jellagen^®^ collagen is free of residual contaminants such as prions, proteins, and polysaccharides. It also brings reduced off-target effects due to its inert, non-specific miRNA. In addition, centuries of conserved evolutionary structure and simple jellyfish physiology ensure that Jellagen ® collagen exhibits excellent batch-to-batch consistency [24]. Another remarkable aspect of jellyfish collagen is its homogeneity with mammalian types I, II, III, V, and IX due to the ancient chemical lineage [19,37]. As a so-called “type 0 collagen”, collagen derived from *Rhizostoma pulmo* in particular is expected to culture every cell type [24,25,28]. 

In this context, jellyfish collagen is of great interest as a cell culture matrix for in vitro cytocompatibility experiments in the field of biomaterials and tissue engineering. It is also intriguing whether the cytocompatibility of Jellagen^®^ collagen can be compared to that of porcine and bovine collagen when co-incubated with fibroblasts and osteoblasts. Therefore, in this study, L929 and MC3T3 cells were co-incubated with the extracts of Jellagen®, porcine, and bovine collagen-coated well plates. XTT, BrdU, and LHD assays, which are also described in the DIN ISO norms 1099-5/-12 for cytocompatibility analyses of medical devices, were used to determine cell viability, multiplication, and cytotoxicity, respectively. As the most used fibroblast for in vitro cytocompatibility, the L929 cells used in this study showed a decreased cell viability in all collagen and control groups when compared to the medium control. It is worth noting that the viability of L929 cells in Jellagen^®^ collagen group was significantly higher in comparison to the bovine and porcine collagen groups. In contrast, both the proliferation and cytotoxicity of all collagen groups were significantly higher than the values in the group of the medium control. However, the Jellagen^®^ collagen coating showed a significantly lower cytotoxicity compared to the porcine and bovine collagen coatings. The above results thus reveal that the Jellagen^®^ collagen coating greatly supports the growth and proliferation of L929 cells as a culture substrate.

In this study, the lower cell viability of L929 cells was conspicuous after co-incubation with extracts of the porcine and bovine collagen coatings for 24h when compared to the other groups. In this context, what triggered these lower values can only be conjectured, as little is known about the exact origin and especially the processing of the collagen. For example, the user does not know which collagen type and which chemicals, or which collagen fibril type, was used for the production of the respective coatings. It is precisely in this area that there is still a need for clarification, since it is known that the different fibrillar precipitation types also cause different cellular responses [17,38]. However, the research community and the manufacturers have only recognized these issues in the last few years, which is why there is little knowledge in this area and why significant research is still needed regarding these issues. In contrast, the porcine and bovine collagen coatings did not affect the metabolic activity of the L929 fibroblasts. The LIVE/DEAD staining data showed that, in contrast to the acute death of most cells in the positive control group (RM-A), the L929 fibroblasts had a spindle-like shape and formed a dense cell layer in all collagen groups. It is worth noting that slightly fewer cells adhered to the porcine collagen coating than to the other collagen coatings. These results appear to be consistent with the study of Böhm et al., which showed that fibroblasts display sparse initial adhesion on both porcine and equine collagen surfaces compared to bovine collagen [39]. This is attributed to the fact that cells adhere to different surfaces at different initial rates. Consideration should also be given to the incomplete adhesion of the cells due to the poor wettability of the coating (cells were thus washed off during the staining process). Altogether, the above results conclude that the Jellagen^®^ collagen from *R. pulmo* is fully biocompatible and is even more recommended than collagen from porcine and bovine sources to be used as a device for the in vitro testing of the cytocompatibility of biomaterials.

The MT3T3 cell line is well-known as a model for studying the cytocompatibility of bone substitute materials as well as the process of osteogenesis in vitro [32,40,41]. The results show that all collagen groups induced a satisfactory cell viability, proliferation, and cytotoxicity, suggesting that all collagen coatings allowed for the excellent growth and proliferation of MC3T3 cells and can be used as an alternative device for in vitro analysis models. Interestingly, the Jellagen^®^-collagen-coated group exhibited a significantly lower proliferation of MC3T3 cells when compared to the porcine- and bovine-collagen-coated groups. Similar results were also reported in a previous in vitro study on Jellagen^®^ coating and jellyfish 3D scaffolds [25]. It is necessary to note that only the 24h post-culture results of the MC3T3 cells with Jellagen^®^-collagen-coated extracts were reported in the present study. However, MC3T3 cells typically exhibit a higher rate of DNA synthesis and a gradual increase in cell numbers during the initial phase of in vitro culture up to 9 days [32]. At this stage, MC3T3 does not express alkaline phosphatase and there is no accumulation of the extracellular collagen matrix [42]. At around day 9, cell proliferation reaches a plateau and growth arrests [32]. Therefore, the cell proliferation data should be considered to be a result of experimental limitations rather than a hasty conclusion that the Jellagen^®^ collagen inhibited MC3T3 proliferation at the initial stage when compared to porcine and bovine collagen.

Moreover, the MC3T3 in the Jellagen^®^-collagen-coated group exhibited a slightly higher cytotoxicity compared to all other groups in the LDH assay. However, these data should not be overestimated as, in the previous study, MC3T3 demonstrated the lowest cytotoxicity on Jellagen^®^ collagen 3D scaffolds, even when compared to the medium control and negative control [25]. This low cytotoxicity can be attributed to the 3D jellyfish scaffold structure, which provides additional orientation for cell growth. Once cells grow into and adhere to the internal pores, the 3D scaffold provides an optimal simulation of the in vivo extracellular matrix (ECM) environment, regulating more complex biological functions, including transcriptional regulation, cell migration, proliferation, and differentiation, by recreating cell–matrix interactions via complicated cellular signals [9,24]. It is also worth mentioning that a combination of three different assays was used to assess the cytocompatibility of collagen coatings at different levels. The results of all tests must therefore be considered together in order to draw reliable conclusions. In this context, Jellagen^®^ collagen is therefore recommended as an alternative culture substrate for MC3T3 cells.

Taken together, the reported results indicate that Jellagen^®^ collagen is expected to be a mainstream source of collagen products in the future. The most notable advantage of jellyfish collagen is its high homology to mammalian collagen, particularly type I, which is the basis for its consideration as an alternative to mammalian collagen [5,21]. Mammalian collagen has long faced difficulties with batch-to-batch inconsistency in laboratory studies, which is critical for reproducibility of experiments. The high batch-to-batch consistency of jellyfish collagen could ameliorate this dilemma and provide more reliable data for studies. In addition, jellyfish collagen provides an alternative source for countries and regions where religious restrictions prevent the use of biological products from porcine or bovine sources. Taken together, this study shows that Jellagen^®^, a jellyfish collagen from *R. pulmo*, has excellent cytocompatibility comparable to porcine or bovine collagen as a culture medium for L929 fibroblasts and MC3T3 pre-osteoblasts. It is recommended as an alternative device for the in vitro cytocompatibility evaluation of biomaterials or medical devices.

## 4. Materials and Methods

### 4.1. Preparation of the Well Plates

The preparation of the well plates was conducted following a specialized protocol [43]. All collagen solutions and coating procedures were comparatively conducted for compatibility of the coatings. Jellagen^®^ (JCP96W) was purchased from Jellagen^®^ Limited, Cardiff, U.K. In brief, the respective collagen was initially combined with acetic acid to obtain a 0.1% (*w/v*) collagen solution and then diluted 10-fold to achieve a concentration of 0.01%. For the coating procedure, a concentration of 6–10 μg/cm^2^ was chosen and the well plates were dried at room temperature for several hours to allow the collagen to bind to the surfaces. After an UV-mediated sterilization, the coated well plates were rinsed with sterile, tissue-culture grade water before introducing the cells and media.

### 4.2. Cell Seeding

All coated well plates and controls were incubated with media at 37 °C and 5% CO_2_ for 72 hours. Blank controls comprised the corresponding media and were subtracted from the results. The extraction of all collagen-coated well plates was carried out according to EN ISO 10993-12:2012. Afterwards, fibroblasts and pre-osteoblasts were seeded into the extracts separately and incubated for 24 h at 37 °C and 5% CO_2_. The extracts were seeded with 1 × 10^4^ cells/100 μl for the in vitro assays, and 2.4 × 10^5^ cells/1 mL for the LIVE/DEAD staining.

### 4.3. Cytocompatibility Analyses

The cytocompatibility analyses were applied in accordance to protocols of the DIN EN ISO 10993-5: 2009/-12: 2012, as previously published [25,44]. In brief, L-929 mouse fibroblasts and MC3T3 pre-osteoblasts, purchased from the European Collection of Cell Cultures, ECACC (Salisbury, U.K.) were used under standard cell culture conditions, i.e., 37 °C, 5% CO_2_, and 95% humidity for 24 h. Blank values that included determination only with a medium but without cells were used as so-called medium controls and were also measured in triplicate determination. These values were additionally subtracted from all other values. RM-A materials, which included polyurethane film with 0.1% zinc diethyldithiocarbamate (ZDEC) (Hatano Research Institute, Food and Drug Safety Center, Hadano, Japan), were used as positive control. Finally, grade 4 titanium plates were applied as a negative control.

Test kits for the analyses of the viability, proliferation, and cytotoxicity were conducted in triplicates, i.e., the Sodium 3,3′-[1(phenylamino)carbonyl]-3,4-tetrazolium]-3is(4-methoxy-6-nitro) Benzene Sulfonic acid Hydrate (XTT)-assay (Roche Diagnostics, Mannheim, Germany), Bromdesoxyuridin (BrdU) ELISA (Roche Diagnostics, Mannheim, Germany), and Lactate Dehydrogenase (LDH) assay (BioVision, Milpitas, CA, USA). All assays were carried out according to the manufacturer’s instructions. The protocols are briefly described as follows. 

XTT assay. Initially, the electron coupling reagent was mixed with the XTT labelling reagent in a ratio of 1:50. A total of 50 μL of this mixture was then added to the cells and incubated for 4 hours under standard cell culture conditions. Afterwards, the absorbance of 100 μL aliquots in a new 96-well plate was assessed by a scanning multi-well spectrophotometer (ELISA reader) with filters for 450 and 650 nm.

BrdU assay. Briefly, the cells were co-incubated with BrdU for 2 hours under cell culture conditions. Following this, FixDenat reagent was used to fix the cells at room temperature. The cells were then treated with anti-BrdU peroxidase (POD) antibody for 1 hour, followed by washing (three times for 5 min). After adding tetramethylbenzidine (TMB) to react with the substrate for 20 min at room temperature, 25 μL 1M H_2_SO_4_ was added to stop the reaction. Finally, absorbance was measured at 450 and 690 nm using a scanning multiwell spectrophotometer (ELISA reader) with a filter.

LDH assay. Briefly, 10 μL of cell supernatant containing LDH was incubated with 100 μL of LDH reactive solution for 30 minutes at room temperature. Afterwards, the absorbance was analysed at 450 and 650 nm by scanning multiwell spectrophotometer (ELISA reader).

### 4.4. LIVE/DEAD-Staining

The LIVE/DEAD-staining procedure was conducted following the corresponding DIN EN ISO protocol, as 2.4 × 10^5^ cells in 1 ml of cell medium were added into each well of a 12-well-plate so that the surface area/medium ratio was 5.65 cm^2^/mL. The staining was applied after 24 h of incubation under standard cell culture conditions, i.e., 37 °C, 5% CO_2_, and 95% humidity, via a standardized kit (Thermo Fisher, Waltham, MA, USA). Directly after the application of the staining, the cells were analysed using an inverted fluorescence microscope (Nikon ECLIPSE Ti-S/L100, Nikon GmbH, Düsseldorf, Germany). Thereby, the cell culture microscope was combined with a filter for the simultaneous detection of red and green fluorescence. The images were taken via a connected digital camera (Axiocam 208 color, Carl Zeiss AG, Oberkochen, Germany).

### 4.5. Statistical Analysis

The statistical analysis was conducted using ANOVA tests combined with Tukey’s multiple comparisons test by means of the GraphPad Prism 9.4 software (GraphPad Software Inc., La Jolla, CA, USA). The resulting differences were stated as significant if the *p*-values were less than 0.05 (* *p* ≤ 0.05) or as highly significant for *p*-values less than 0.01 (** *p* ≤ 0.01) or less than 0.001 (*** *p* ≤ 0.001). The data were finally graphed as mean values ± standard deviations.

## 5. Conclusions

Stable and reliable 2D and 3D in vitro cell culture models are important preliminary assessment tools for the cytocompatibility of biomaterials. Collagen, a key regulator of cell growth and proliferation, has proven to be an ideal substrate for cell culture. Marine organisms represent a very attractive source of collagen. Particularly, collagen products derived from *R. pulmo* have been shown to support cell growth, adhesion, and interaction. Its potential as a culture substrate for human iPSC-derived microglia and ovarian cancer cells has been demonstrated.

This study provides preliminary results comparing well plates coated with Jellagen^®^ collagen and mammalian collagen (porcine and bovine) as in vitro cell culture devices for the analysis of the cytocompatibility of medical devices with fibroblasts and osteoblasts. Both L929 fibroblasts and MC3T3 pre-osteoblasts showed significantly higher cell viability after co-incubation with extracts of Jellagen^®^ collagen coating when compared to that of porcine and bovine collagen coatings. Both cells also showed satisfactory proliferation and cytotoxicity in the group with Jellagen^®^ collagen coating. L929 cells also formed the densest cell layer on the Jellagen^®^ collagen coating and demonstrated healthy behaviour. Overall, Jellagen^®^ collagen is a fully biocompatible cell culture substrate suitable for in vitro cytocompatibility analyses in the field of biomaterials and tissue engineering.

## Data Availability

All relevant data are included in the manuscript.
